# Genetic, Morphological and Antigenic Relationships between Mesonivirus Isolates from Australian Mosquitoes and Evidence for Their Horizontal Transmission

**DOI:** 10.3390/v12101159

**Published:** 2020-10-13

**Authors:** Natalee D. Newton, Agathe M. G. Colmant, Caitlin A. O’Brien, Emma Ledger, Devina Paramitha, Helle Bielefeldt-Ohmann, Daniel Watterson, Breeanna J. McLean, Sonja Hall-Mendelin, David Warrilow, Andrew F. van den Hurk, Wenjun Liu, Christina Hoare, Joanne R. Kizu, Penelope J. Gauci, John Haniotis, Stephen L. Doggett, Babak Shaban, Cheryl A. Johansen, Roy A. Hall, Jody Hobson-Peters

**Affiliations:** 1 School of Chemistry and Molecular Biosciences, University of Queensland, St Lucia, Queensland 4072, Australia; natalee.newton@uq.edu.au (N.D.N.); agathe.colmant@uq.net.au (A.M.G.C.); caitlin.obrien@uqconnect.edu.au (C.A.O.); emma.ledger@uq.net.au (E.L.); devina.paramitha@uq.net.au (D.P.); h.bielefeldtohmann1@uq.edu.au (H.B.-O.); d.watterson@uq.edu.au (D.W.); breeanna.mclean@monash.edu (B.J.M.); roy.hall@uq.edu.au (R.A.H.); 2 Australian Infectious Diseases Research Centre, University of Queensland, St Lucia, Queensland 4072, Australia; 3 School of Veterinary Science, University of Queensland Gatton Campus, Gatton, Queensland 4343, Australia; 4 Public Health Virology, Queensland Health Forensic and Scientific Services, Brisbane, Queensland 4108, Australia; Sonja.Hall-Mendelin@health.qld.gov.au (S.H.-M.); David.Warrilow@health.qld.gov.au (D.W.); andrew.vandenhurk@health.qld.gov.au (A.F.v.d.H.); 5 Australian Defence Force Malaria and Infectious Disease Institute, Gallipoli Barracks, Enoggera, Queensland 4051, Australia; wenjun.liu@defence.gov.au (W.L.); Christina.hoare@defence.gov.au (C.H.); Joanne.kizu@defence.gov.au (J.R.K.); 6 Defence Science & Technology Group, Fisherman’s Bend, Victoria 3207, Australia; Penny.Gauci@dst.defence.gov.au; 7 NSW Health Pathology-ICPMR Westmead, Medical Entomology, Westmead Hospital, Westmead 2145, Australia; John.Haniotis@health.nsw.gov.au (J.H.); Stephen.Doggett@health.nsw.gov.au (S.L.D.); 8 Australian Genome Research Facility Ltd., Parkville 3050, Australia; bshaban@unimelb.edu.au; 9 School of Pathology and Laboratory Medicine, Nedlands 6009, Australia; cheryl.johansen@health.wa.gov.au; 10 PathWest Laboratory Medicine, Nedlands 6009, Australia

**Keywords:** mesonivirus, nidovirus, insect-specific virus, monoclonal antibody, FTA card

## Abstract

The *Mesoniviridae* are a newly assigned family of viruses in the order *Nidovirales*. Unlike other nidoviruses, which include the *Coronaviridae*, mesoniviruses are restricted to mosquito hosts and do not infect vertebrate cells. To date there is little information on the morphological and antigenic characteristics of this new group of viruses and a dearth of mesonivirus-specific research tools. In this study we determined the genetic relationships of recent Australian isolates of Alphamesonivirus 4 (Casuarina virus—CASV) and Alphamesonivirus 1 (Nam Dinh virus—NDiV), obtained from multiple mosquito species. Australian isolates of NDiV showed high-level similarity to the prototype NDiV isolate from Vietnam (99% nucleotide (nt) and amino acid (aa) identity). Isolates of CASV from Central Queensland were genetically very similar to the prototype virus from Darwin (95–96% nt and 91–92% aa identity). Electron microscopy studies demonstrated that virion diameter (≈80 nm) and spike length (≈10 nm) were similar for both viruses. Monoclonal antibodies specific to CASV and NDiV revealed a close antigenic relationship between the two viruses with 13/34 mAbs recognising both viruses. We also detected NDiV RNA on honey-soaked nucleic acid preservation cards fed on by wild mosquitoes supporting a possible mechanism of horizontal transmission between insects in nature.

## 1. Introduction

The order *Nidovirales* includes four genetically diverse families—*Coronaviridae*, *Arteriviridae*, *Roniviridae* and *Mesoniviridae*. Nidoviruses are enveloped viruses containing positive-sense single-stranded RNA (+ssRNA) genomes ranging in length from ≈12 to 31 kb [1,2]. Some nidoviruses are human pathogens (e.g., SARS-CoV-1, SARS-CoV-2 and MERS), while others are pathogens of other mammals, birds, fish and crustaceans.

*Mesoniviridae* represent a unique nidovirus family due to the restriction of their replication to only insects [3,4]. The members of the type species, Alphamesonivirus 1, include Nam Dinh virus (NDiV), isolated from mosquitoes captured in Vietnam in 2004 [5] and the independently discovered Cavally virus (CavV) from Côte d’Ivoire (2004) [6]. Eight additional mesonivirus species that have been isolated from mosquitoes of various genera, have since been classified (Alphamesonivirus 2–Alphamesonivirus 9) [7]. Casuarina virus (CASV), the first Australian mesonivirus was classified as a discrete species named Alphamesonivirus 4 [3]. We believe that additional phenotypic studies, including virion morphology and antigenic structure are required to support these classifications.

Mesoniviruses do not infect vertebrates and have no known association with human or animal disease. However, they are of growing interest due to their relationship to SARS-CoV-2 and other emerging coronavirus pathogens. Indeed, the size of their genome (20–21 kb) provides an evolutionary link between small nidoviruses (12.7–15.6 kb) such as the arteriviruses and large nidoviruses such as the coronaviruses and roniviruses (26.3–31.6 kb) [5,8]. The mesonivirus genome contains seven major open reading frames (ORFs), with the exception of Méno virus (MénoV), which only has six [3,4,5,6,8]. The two largest ORFs, ORF1a and ORF1b, are located at the 5’ end of the genome and overlap by a few nucleotides, with translation regulated by a ribosomal frameshift motif (RFS) allowing the two respective polyproteins, pp1a and pp1ab, to be expressed [1,6,9]. ORFs1a and 1b code for the key nidovirus-conserved replicative enzymes. These key enzymes include the viral 3C-like chymotrypsin-like protease encoded by ORF1a and the RNA-dependent RNA polymerase (RdRp), helicase (Hel), exoribonuclease (ExoN), N7-methyl-transferase (NMT) and 2’-O-methyltransferase (OMT) encoded by ORF1b [4]. The other five major ORFs are translated from subgenomic mRNAs encoding structural and accessory proteins [6]. These structural proteins include the glycoprotein spike (S protein, S1 and S2 subunits) encoded by ORF2a, the nucleocapsid protein (N) (ORF2b), and membrane-spanning proteins (M) encoded by ORF3a [4].

The structural proteins of mesoniviruses are predicted to have similar functions as other nidoviruses. The spike protein (S) is a class I fusion protein that exists as a homotrimer on the viral envelope [3,10]. The S protein consists of two subunits, S1 and S2. S1 forms the globular structure and commonly contains neutralising epitopes as it is responsible for binding to host cell receptors [3,10]. The S2 subunit comprises the stalk section of the S protein and mediates fusion of the virus to the host cell membrane. The M protein is necessary for virion budding and provides essential interaction between N and S proteins whereas, the N proteins are involved in packaging and encapsidation of the genome [11].

There is growing evidence that the maintenance of insect-specific viruses within mosquito populations is via vertical transmission [12,13,14,15]. Indeed, the detection of mesoniviruses in adult male and female mosquitoes supports the hypothesis that mesoniviruses are transmitted from the female mosquito to her progeny [16,17], or via sexual transmission. However, recent studies also provide evidence that horizontal transmission of mesoniviruses is plausible, with the detection of mesonivirus viral RNA in the saliva of infected mosquitoes [18], as well as on honey-baited cards, upon which mosquitoes expectorate saliva [16,19]. These data suggest several potential routes of mesonivirus transmission between mosquitoes.

In this study we used transmission electron microscopy to investigate the comparative virion morphology of Australian isolates of CASV and NDiV. We also used a panel of monoclonal antibodies (mAbs) produced to CASV and NDiV to explore the antigenic relationships of these viruses. Our detection of NDiV RNA using a novel method for monitoring viral transmission in mosquito saliva in the field also provides further evidence for a mechanism of horizontal transmission of mesoniviruses.

## 2. Materials and Methods

### 2.1. Cell Culture and Virus Isolation

*Aedes albopictus* C6/36 cells were maintained in RPMI media supplemented with 5% fetal bovine serum 50 μg/mL streptomycin, 50 U/mL penicillin and 2 mM L-glutamine at 28 °C. Viruses were propagated in C6/36 cells with an incubation period of 2–5 days. Virus titres were established using a 50% tissue culture infective dose (TCID_50_) assay [20,21].

### 2.2. Detection and Isolation of Mesoniviruses from Homogenised Mosquito Pools

Mosquito homogenates were screened for the presence of viruses as previously described [22,23]. Adult mosquitoes were collected using CO_2_-baited light traps from a number of locations throughout Australia (Figure S1). Briefly, the mosquitoes were homogenised and filtered prior to inoculation onto monolayers of C6/36 cells and incubated at 28 °C for 5–7 days. The culture supernatant was retained and the cell monolayers fixed with a solution of 20% acetone in phosphate buffered saline (PBS) with 0.02% bovine serum albumin at 4 °C overnight. ELISA was performed on the fixed cells using mAbs 3G1 and 2G4 (named MAVRIC [22]). For screening of homogenates from Darwin and Western Australia, anti-mesonivirus mAbs generated in this study were used. RNA was extracted from ELISA-positive samples and assessed for the presence of mesoniviruses using RT-PCR as detailed below, followed by Sanger sequencing at the Australian Genome Research Facility (AGRF, Brisbane, Australia).

### 2.3. Genome Sequencing and Phylogenetic Analysis

NDiV isolates DC59899, DC60042, DC59801 and 179853 were cultured and RNA extracted using the Macherey-Nagel (Duren, Germany) NucleoSpin Viral RNA isolation kit as per the manufacturer’s instructions. Deep sequencing was performed using Illumina (CA, USA) on a HiSeq2000 at the Australian Genome Research Facility. Sequence data for 179853 was first analysed by de novo assembly using Velvet Assembler (https://www.ebi.ac.uk/~zerbino/velvet/) followed by elongation of assembled sequences using Oases. The resulting contigs were run through tBlastx which identified sequence with high similarity to NDiV. Following this, the full genome sequence of NDiV 179853 was assembled by mapping paired reads to NDiV Houston strain (accession KC807176.1) in Geneious v8.1.4 (Auckland, New Zealand). The genomes of the remaining NDiV isolates were assembled by mapping the paired reads against Genbank accession number NC_015874.1 in Geneious v8.1.4. For the Shoalwater Bay Training Area (SWBTA) CASV isolates, total viral RNA was extracted from a 140 µL of passage 2 tissue culture supernatant using RNeasy-Mini kit (Qiagen Germany), prior to being converted to cDNA using the REPLI-g WTA Single Cell kit (Qiagen). The cDNA library was prepared using the Nextera XT Kit (Illumina, CA, USA) and sequenced on a MiSeq instrument using a Reagent Micro Kit v2, 300-cycles (Illumina) performed according to the standard protocols to produce approximately a million reads (2 × 150 nt) for each sample. The sequence data were assembled by de novo assembly and blast with existing sequences in GenBank using Geneious R11 (version 11.1.2) software with default parameters setting for initial analysis. The Blastn results showed similarity to CASV (GenBank accession number NC_023986). The MiSEQ sequence data were re-mapped to CASV reference genome (GenBank accession number NC_023986) to generate consensus sequences. The 5’UTR sequences of two identified virus (SWBTA-Ann and SWBTA-vigilax) were determined by Sanger sequencing of the reverse transcription and PCR amplicons using 5’/3’ rapid amplification of cDNA ends (RACE) kit (Roche, Mannheim, Germany) according to the manufacturer’s instructions.

Muscle alignments were performed on available nucleotide sequences for mesonivirus genomes that were coding-complete. The alignments were used to construct phylogenetic trees using MrBayes v3.2.2 under the Bayesian Marko chain Monte Carlo (MCMC) model with General Time Reversible (GTR) substitution model, gamma distribution (five discrete gamma categories) and invariant rates among sites [24] in Geneious 8.1.9.

### 2.4. Electron Microscopy—Purification and TEM

The virion morphology of CASV (strain 0071 [3]) and NDiV (strain 179853) was analysed by transmission electron microscopy (TEM) as previously described [3]. Briefly, the virions were concentrated from culture supernatant with PEG 8000, resuspended in NTE buffer (12 mM Tris pH8, 120 mM NaCl, 1 mM EDTA pH 8) before ultracentrifugation through a 20% sucrose cushion and 10–40% potassium tartrate gradient. The virus band was harvested and buffer-exchanged into NTE, prepared for TEM on a formvar-coated copper grid and negatively stained with 1% uranyl acetate. Virions were visualised on a JEOL1010 transmission electron microscope. The length of the spikes protruding from the surface of the mesonivirus virions were measured using ImageJ.

### 2.5. Preparation of Mouse Antiserum and Monoclonal Antibodies to CASV

Hybridomas secreting monoclonal antibodies specific to CASV or NDiV were derived from BALB/c mice immunised with purified virions as previously described under Animal Ethics Committee approval number SCMB/AIBN/329/15/ARC [25]. Hybridomas were maintained at 37 °C 5% CO_2_ in hybridoma serum-free media supplemented with 50 μg/mL streptomycin, 50 U/mL penicillin and 2 mM L-glutamine with 20% FBS initially and then weaned to growth in serum-free media. Isotyping of the mAbs was performed using the Mouse Monoclonal Antibody Isotyping Reagent (Sigma Aldrich Australia, Castle Hill, NSW, Australia) as per the manufacturer’s instructions. The reactivity of each mAb to CASV or NDiV antigen was assessed by ELISA as previously described, using fixed CASV and NDiV-infected C6/36 cell monolayers [23,25]. Select monoclonal antibodies described in this study are available from Mozzy mAbs (https://eshop.uniquest.com.au/mozzy-mabs/).

### 2.6. SDS-PAGE, Western Blot and Immunoprecipitation

Lysates of virus-infected C6/36 cells were prepared by incubating monolayers with 3.5 mL/10^7^ cells of NP-40 lysis buffer (1% NP-40, 50 mM Tris-Cl, 150 mM NaCl, 2 mM EDTA, pH 7.5; 1/1000 Sigma protease inhibitor) at 4 °C for 30 min. Lysate was clarified by centrifugation and the supernatant stored at −80 °C. The lysate was resolved on a 4–12% Bis-Tris SDS-PAGE gel before transferring onto a nitrocellulose membrane. The membrane was blocked in TENTC blocking buffer (0.05 M Tris/HCl (pH 8.0), 1 mM EDTA, 0.15 M NaCl, 0.05% (*v*/*v*) Tween 20, 0.2% *w*/*v* casein), before probing with the appropriate antibody as hybridoma culture supernatant for 1 h. After washing with TBST (tris-buffered saline, 0.05% Tween-20), the membrane was incubated for 1 h with IR Dye-conjugated goat anti-mouse IgG (IRDye 800 CW goat anti-mouse IgG, LI-COR Biosciences, Lincoln, NE, USA). After a final wash, the membrane was imaged with the Odyssey imaging system (LI-COR). Immunoprecipitation pulldowns were used to test antibodies that did not bind in Western blot as described previously [14]. Briefly, 60 μL of Protein G beads (Dynabeads; Thermo Fisher Scientific, Scoresby, Vic, Australia) were washed 1× with PBST before incubating with hybridoma culture supernatant on a rotary shaker for 1 h at room temperature. Beads were washed 3× with PBST and once with NP-40 lysis buffer before incubation with viral lysate for 1 h with mixing. After washing, the protein was eluted with 0.1 M Glycine for 8 min with mixing before neutralisation in 1 M Tris. Samples were visualised by SDS-PAGE and Sypro Ruby stain as previously described [23].

### 2.7. Microneutralisation Assay

A microneutralisation assay was performed as per previously described methods with modifications [14,23,26]. Briefly, serial tenfold dilutions were performed on mAb or polyclonal sera (starting undiluted or at 1/10) in RPMI media before adding 100 TCID_50_ units per well of virus and incubating for 1 h at 28 °C. C6/36 cells were added at 1 × 10^4^ cells per well (of a 96-well plate) and incubated for 3 days at 28 °C. Observations of CPE were noted and plates were fixed with acetone fixative buffer (20% acetone in phosphate buffered saline (PBS) with 0.02% bovine serum albumin), after which a fixed cell ELISA was performed with (anti-dsRNA mAb 3G1 [22] or for CASV assays, C.9D7). The neutralising titre was determined as the highest dilution for which both duplicate wells had no detectable virus.

### 2.8. RT-PCR

Viral RNA was extracted from culture supernatant using the Macherey-Nagel (Düren, Germany) Viral RNA isolation kit as per the manufacturer’s instructions. RT-PCR was performed using Invitrogen Superscript III One-step RT-PCR system with Platinum Taq DNA polymerase and a generic mesonivirus primer set NidoF1: 5′-GTTGTATGCTATGCCGYCG-3′, NidoR1: 5′-TCCATAGTATCGTAGCAATTCC-3′ and the following cycling conditions; reverse transcription: 45 °C/30 min; PCR: one cycle 94 °C/2 min; 40 cycles 94 °C/30 s, 45 °C/30 s, 68 °C/1 min; followed by a final extension cycle of 68 °C/5 min. PCR products were confirmed by excising under UV and DNA extracted using Macherey-Nagel NucleoSpin Gel and PCR Clean-up as per manufacturer’s instructions. Samples were submitted for Sanger sequencing at AGRF.

### 2.9. Viral RNA Extraction from Mosquito Saliva on Flinders Technology Associates (FTA) Cards

Mosquito traps (CO_2_-baited updraft box traps) were deployed fortnightly in various locations throughout Queensland. These traps were designed to enable wild mosquitoes to feed on honey-soaked FTA cards, onto which infected mosquitoes expectorate saliva containing virus. This system is routinely used for arbovirus surveillance in Queensland [27,28]. FTA cards were collected from the traps and sent to the Public Health Virology laboratory in Brisbane (Queensland Health, Forensic and Scientific Services) for processing. Viral RNA was eluted and extracted from cards using the QIAamp^®^ Virus BioRobot^®^ MDx Kit (Qiagen, Clifton Hill, Australia) with the Bio Robot Universal System (Qiagen, Hilden, Germany) according to manufacturer’s instructions [28]. RT-PCR was performed on the extracted RNA as above using F: 5′-CACACTCCATCTCCACAGCACACC-3′. R: 5′-TATGCAAAAGCAAGCCGAATTC AA-3′ and the cycling conditions of 45 °C/30 min, 94 °C/2 min; followed by 40 cycles of 94 °C/30 s, 53 °C/30 s, 68 °C/30 s, and final extension for 68 °C/5 min.

### 2.10. Deep Sequencing of Viral RNA Eluted from FTA Cards

Libraries were prepared as described previously [29]. Briefly, residual DNA was removed from the RNA preparation using the Heat and Run kit (ArcticZymes Technologies ASA, Tromsø, Norway) which contains a heat-labile DNase. First strand cDNA was prepared using the Protoscript II kit (New England Biolabs, Ipswich, MA, USA), followed by second strand synthesis using a cocktail of RNase H, *E. coli* DNA ligase and DNA polymerase I (New England Biolabs). cDNA libraries were then constructed using the Nextera XT system (Illumina) and sequenced on a NextSeq 500 using a v2 mid-output kit, and 3.8 M total reads (2 × 150 nt paired) were generated. Metagenomic analysis of the data was performed by standalone Blastn with default parameters and observing the output with MEtaGenomic ANalyser (MEGAN v6.15.2) software [30]. Coverage of the genome was determined by reference assembly using Geneious (v11.1.5) [31] and the NDiV genome as a reference (GenBank accession number NC_015874.1).

## 3. Results

### 3.1. Isolation of Two Mesoniviruses from Multiple Mosquito Species in Australia

Prior to this study, only a single mesonivirus isolate, CASV (Alphamesonivirus 4), obtained from *Coquillitidia xanthogaster* mosquitoes collected in Darwin, Northern Territory, had been described from Australia [3]. To further assess the prevalence of mesoniviruses within Australian mosquitoes, 568 mosquito pools, from different geographical regions of Australia, were screened for viruses by inoculation of the mosquito homogenates onto mosquito cell monolayers and detection of replicating RNA viruses via the presence of dsRNA in ELISA (Figure S1) [22]. RNA extracted from culture supernatants for those samples that were positive for dsRNA, were further analysed by RT-PCR using mesonivirus-specific primers, or sent directly for deep sequencing (Shoalwater Bay isolates). These mosquito pools included a range of species from *Culex*, *Aedes*, *Coquillitidia* and *Anopheles* genera. A total of 79 mesonivirus isolates were obtained; 11 isolates of CASV from *Culex annulirostris*, *Aedes vigilax* and *Aedes procax* collected in northern Queensland (QLD), and 68 isolates of NDiV (Alphamesonivirus 1) from a wide variety of species collected in Western Australia (WA), New South Wales (NSW) and QLD (Table 1). The samples which represented the greatest mosquito species diversity were from WA, which also had the highest detection of NDiV (62%, 56 out of 91 pools screened). These represent the first isolates of NDiV (Alphamesonivirus 1) in Australia and the first report of CASV (Alphamesonivirus 4) in *Cx. annulirostris*, *Ae. vigilax* and *Ae. procax*.

The complete ORF of four NDiV isolates from different mosquito species, was elucidated via next generation sequencing (WA isolates—DC59899 accession number MT514349, DC59801 accession number MT514350, DC60042 accession number MT514348; Qld isolate 179853 accession number MT514351). Three Qld CASV isolates were also sequenced. Of these, full length sequence was obtained, including the UTRs, for the isolates derived from *Ae. vigilax* (accession number MT522183) and *Cx. annulirostris* (accession number MT522182). Next generation sequencing was also performed on the *Ae. procax* isolate (accession number MT522184), of which 198 reads were derived that mapped to CASV.

**Figure 1:**
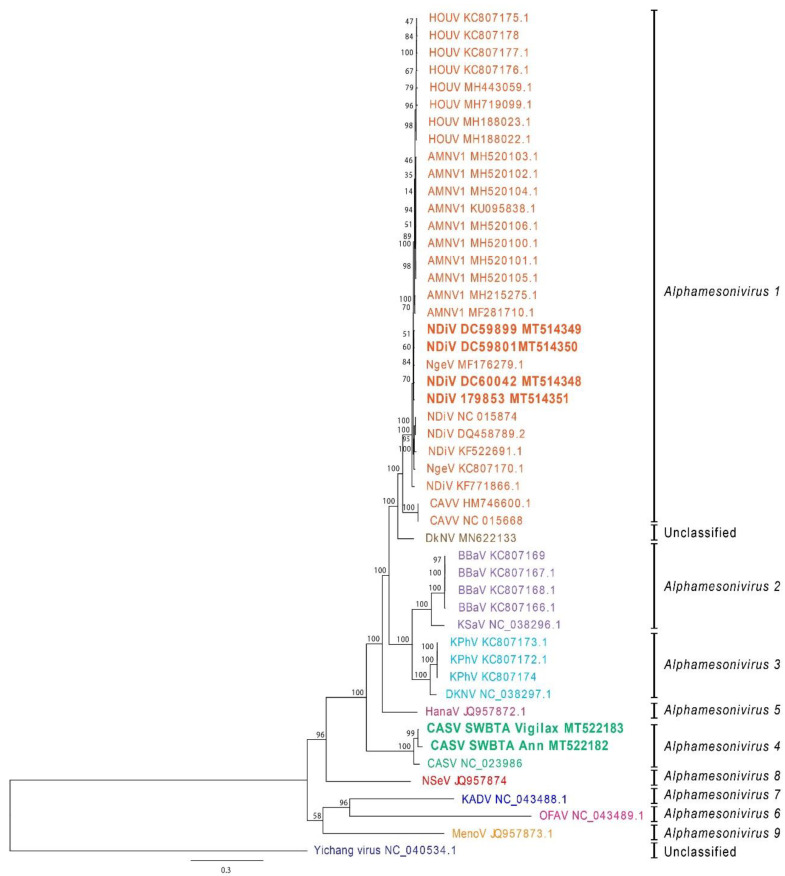
Phylogenetic analysis of the new mesonivirus sequences with other members of the *Mesoniviridae*. The tree was constructed in Geneious (Auckland, New Zealand) using MrBayes v3.2.2 under the Bayesian Marko chain Monte Carlo (MCMC) model with General Time Reversible (GTR) substitution model, gamma distribution (five discrete gamma categories) and invariant rates among sites [24] using available nucleotide sequences for virus genomes that were coding-complete. Horizontal branch lengths represent posterior probabilities. Bolded text indicates new Australian Alphamesonivirus 1 isolates (179853 (NSW), DC59899 (WA), DC59801 (WA), DC60042 (WA)) and Alphamesonivirus 4 isolates (SWBTA Vigilax and SWBTA Ann, both from Qld.). Scale bar indicates substitutions per site.

To determine the genetic relationship of the new isolates of NDiV and CASV with the prototype isolates of each species, an unrooted phylogenetic tree was constructed based on complete ORF sequences (Figure 1). As expected, and in agreement with published phylogenetic studies, this phylogenetic tree shows CASV and NDiV as discrete sister clades [3]. NDiV isolates from NSW and WA (bolded) clustered with the prototype NDiV isolate (NC_015874) and the CASV isolates from Shoalwater Bay clustered with the prototype CASV isolate. Each of the Australian NDiV isolates shared a 99% nucleotide and amino acid identity across the ORF1ab with the prototype NDiV, whereas the Shoalwater Bay CASV isolates shared 95–96% nucleotide and 90–91% amino acid identity with the prototype CASV (Table 2). Comparisons over the entire ORF returned the same nucleotide and amino acid identities.

### 3.2. Comparison of Virion Morphology of CASV and NDiV by Electron Microscopy

**Figure 2:**
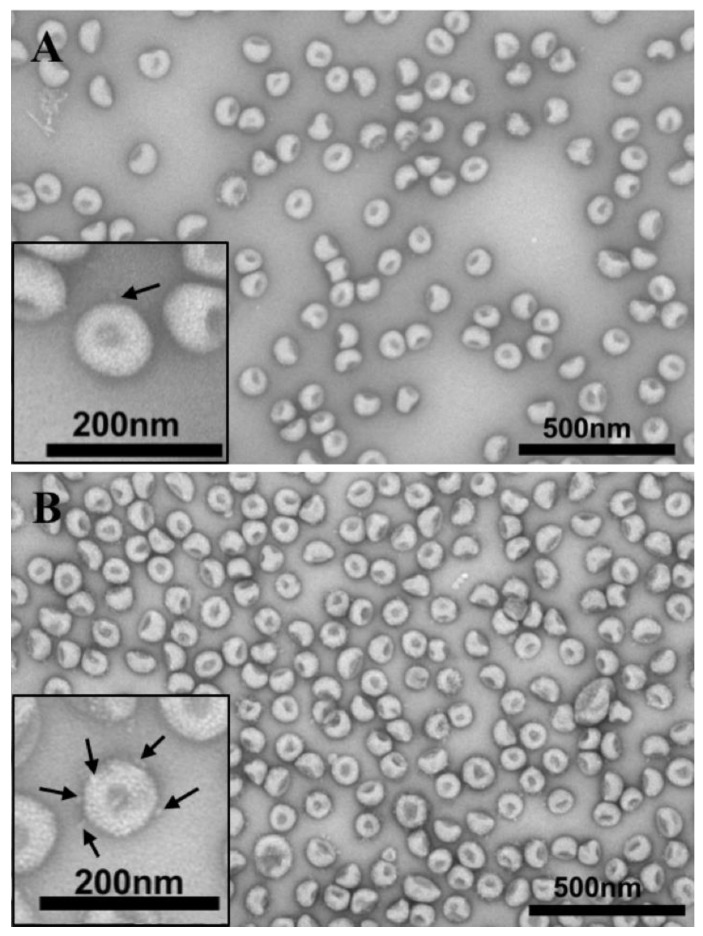
Transmission electron microscopy of CASV-0071 and NDiV-179853. Electron micrograph of potassium tartrate gradient-purified CASV (**A**) and NDiV (**B**) virions following staining with 1% uranyl acetate. Spike protrusions marked with arrows. Scale bar indicated.

Previously, variable spike lengths have been reported for the virions of Alphamesonivirus 1 [4,32]. The virion morphology of one of the Australian NDiV isolates (179853) was analysed and compared to purified particles of CASV (2010 Darwin prototype isolate 0071) via transmission electron microscopy. Virions of uniform size were observed at approximately 80 nm for both CASV and NDiV, with the spike structures protruding from the outside of the virion (Figure 2). Spikes measured approximately 10 nm for both CASV and NDiV.

### 3.3. Production of Monoclonal Antibodies to CASV and NDiV

Monoclonal antibodies were produced to purified preparations of CASV-0071 and NDiV-179853 as previously described [26]. Eight mAbs for CASV and 26 mAbs for NDiV were obtained for each fusion. The specificity of each mAb was determined by fixed-cell ELISA (Table 3). Three of the CASV-derived mAbs cross-reacted with NDiV, while 10 NDiV-derived mAbs cross-reacted with CASV (Table 3). Isotyping of each mAb revealed a variety of isotypes including IgG_1_, IgG_2a_, IgG_2b_, and IgM. One mAb (C.10H11) was isotyped as IgM and IgG_2a_, suggesting possible class switching, or a non-clonal hybridoma. All CASV-derived mAbs that cross-reacted with NDiV were identified as IgM.

The protein target for each mAb was determined by Western blot or immunoprecipitation. Of the CASV-derived cohort, four bound the M protein, while two recognised N and two recognised S (Table 3, Figures S2 and S3). Of the NDiV-derived mAbs for which the protein target could be elucidated, five bound M, two bound S and three bound N. For both suites of antibodies, only those that bound M were reactive to both CASV and NDiV (Table 3 and Table S1). The protein target of an additional five mAbs that bound both CASV and NDiV could not be elucidated by Western blot.

To determine whether each mAb had the ability to neutralise mesoniviruses in C6/36 cells, a microneutralisation assay was conducted. As expected, those mAbs that returned high neutralising titres were all directed to the spike protein. Of those mAbs that had neutralising capacity, they could only neutralise the virus to which they were derived (Table 3).

**Figure 3:**
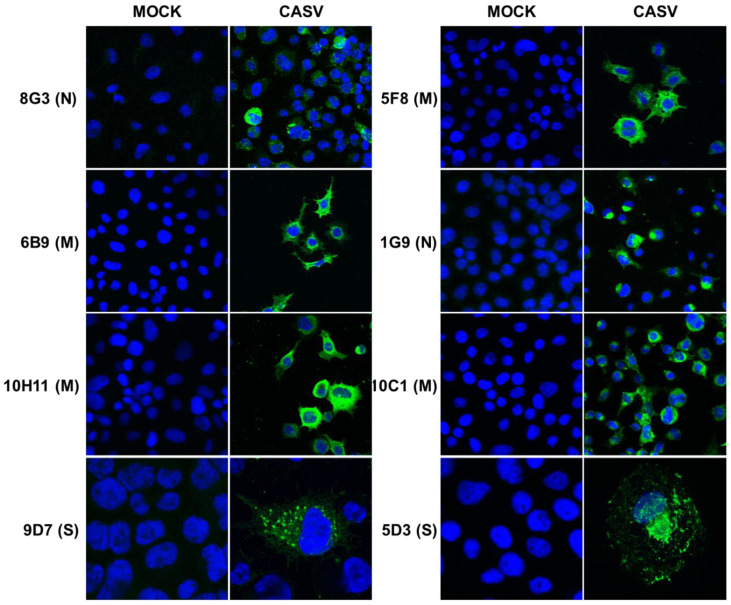
IFA analysis by confocal microscopy of CASV mAbs. Antibodies recognising each viral protein (S, M and N) were used to stain fixed CASV-infected C6/36 cells. Viral antigen in green and cell nuclei were stained with Hoechst 33342 in blue. Slides were imaged at 20× magnification, panels 5D3 and 9D7 imaged at 63×.

The CASV-derived mAbs were also shown to detect viral antigen by IFA in CASV-infected C6/36 cells (Figure 3). The anti-N mAbs displayed strong perinuclear staining, as too did those mAbs directed to the M protein. However, in some cases, the M-reactive mAb labelling also extended diffusely into the cell cytoplasm. The anti-spike protein mAbs facilitated the observation of spike protein labelling throughout the cellular secretory pathway (Figure 3).

### 3.4. Detection of Mesonivirus RNA in Mosquito Saliva during Routine Arbovirus Surveillance

Given the high prevalence of mesoniviruses in different regions of Australia and their presence in multiple mosquito species, we further assessed if these viruses could be detected in mosquito saliva collected on honey-soaked FTA cards. To achieve this, we sampled RNA eluted from specialised traps deployed in a number of locations in Qld and NT. RNA eluates from 57 FTA cards collected were screened by RT-PCR with primers that targeted a 75 bp region in the RdRp of both NDiV and CASV. A total of seven samples were positive for mesoniviruses from three locations: Emerald (QLD), Longreach (QLD) and Darwin (NT) (Table 4, Figure S1). The species of the virus could not be confirmed by Sanger sequencing, so the RNA extracted from an FTA card deployed in Longreach in 2013 was submitted for next generation sequencing. A total of 244 reads matching the NDiV genome were identified in this sample (Figure S4).

## 4. Discussion

Here, we report the first isolation and characterisation of NDiV (Alphamesonivirus 1) in Australia, and the detection of new CASV (Alphamesonivirus 4) isolates. Using these isolates, we performed structural and antigenic analyses by assessing the virion morphology of both viruses by electron microscopy, and generating and characterising novel monoclonal antibodies. We also report the detection of NDiV RNA from FTA cards, presumably from expectorated saliva from wild mosquitoes.

The first isolates of NDiV, the prototype mesonivirus, originated from *Culex* mosquitoes collected in Vietnam in 2002 [5]. Strains of this virus were subsequently isolated from various locations around the world (reviewed in [33]), including the detection of Alphamesonivirus 1 (strain Ngewotan virus) RNA in *Cx. australicus* collected in Western Australia in 2015 [34]. Herein, we report the isolation of NDiV from mosquitoes collected over an eight year period (2007–2014) and from a wide range of mosquito species and genera within Australia. The virus was isolated from both the east and west coasts of Australia (Peel Region—WA, Darwin—NT, Brisbane—Qld and Ballina—NSW), separated by over 4500 km. Sequence comparisons and phylogenetic analyses clearly indicate that the new Australian NDiV isolates should be classified as strains of Alphamesonivirus 1, due to the high nucleotide identity with other virus strains of this species.

Unlike the vast geographic distribution of Alphamesonivirus 1, CASV (Alphamesonivirus 4) has only been detected in mosquitoes from Australia. Prior to this study, there had been only a single isolation of CASV from *Cq. xanthogaster* mosquitoes collected in Darwin, NT, in 2010. In our study, eight additional isolates were identified from *Cx. annulirostris* mosquitoes collected between 2005 and 2007 in Cairns, Qld, 1700 km away from Darwin. Single isolates were obtained from *Cx. annulirostris*, *Ae. vigilax* and *Ae. procax* collected at Shoalwater Bay, 773 km south of Cairns. Consistent with the trend observed for NDiV, these new CASV isolates still displayed 95–96% nucleotide identity with the prototype strain, despite being isolated from different mosquito genera.

Morphological comparisons of CASV and NDiV virions by TEM were performed using simultaneously purified virions. Minimal differences were observed between the viruses with the overall virion diameter measuring 80 nm, with spike protrusions measuring 10 nm. Our observations for spikes of 10 nm for the NSW isolate of NDiV contrasts the observations of two independent studies whereby spike measurements of 3–4 nm were recorded from the imaging of ultrathin sections, or purified particles [32,35]. The differences may be attributable to variances in electron microscopy procedures, or virion harvesting times post-infection. The size of the prototype NDiV virions have been reported between 40 and 80 nm depending on the staining technique used, or whether the virions were observed in ultrathin sections of glutaraldehyde-fixed virus-infected cells, or if the virus was derived from culture supernatant [32,35]. Such size differences may also reflect the maturity of the particle. The sizes of the CASV virions observed in this study were consistent with those reported previously (diameter of 80 nm this study; 84 nm [3]). At 10 nm, the CASV spike length reported herein is smaller than the 15 nm reported previously. However, this size difference could be attributed to the differences in imaging (cryo-EM previously vs. TEM here). Cryo-EM allows imaging at near-physiological conditions as well as native hydration and does not require the particles to be fixed and stained [36].

The generation of eight mAbs to CASV and 26 to NDiV provide the first mAbs specific for mesoniviruses. The majority of the mAbs targeted the M protein, which is consistent with this protein being an immunodominant structural protein [37]. The three CASV-derived mAbs that cross-reacted to NDiV were also M protein-reactive, suggesting conservation of major antigenic sites on this protein between CASV and NDiV. Similarly, of the NDiV-derived mAbs, only those definitively confirmed as M protein-reactive cross-reacted to CASV. In silico analysis by Zirkel et al. [4] showed that the M protein is genetically closer (70.8%) between the mesoniviruses (CavV, NDiV, NséV, HanaV) compared to N (62.63%) and S (67.4%), providing a possible explanation for the conservation of antigenic sites within the M protein. However, in a direct comparison between the protein sequences of NDiV and CASV, both the M and S proteins share 77% amino acid identity in contrast to only 69% amino acid identity over the N protein. In the context of the mAbs described herein, it should also be noted, however, that 54% of the cross-reactive mAbs generated in this study exhibited an IgM isotype, which can be more prone to cross-reactivity and frequently display differences in epitope affinities in comparison to their IgG counterpart [38,39].

Antigenic homology within the *Mesoniviridae* has been assessed previously using polyclonal antisera raised against synthetic peptides of CavV and NseV proteins [4]. These studies demonstrated antigenic similarity exists for all structural proteins of CavV, NséV, MenoV and HanaV using the CavV/NséV antiserum. However, this wide cross-reactivity could be attributed to the serum being produced by immunisation of mice with viruses from two different mesonivirus subgenera (CavV—Alphamesonivirus 1, subgenus *Namcalivirus*, NseV—Alphamesonivirus 8, subgenus *Menolivirus*). In this study, most anti-CavV structural protein antisera reacted with all viruses tested (CavV, NséV, MenoV, HanaV). These findings combined with our production of anti-M mAbs that bind both CASV and NDiV indicate the presence of conserved antigenic sites within and between the subgenera. Testing of other mesonivirus species with our panel of mAbs will further assist in elucidating antigenic similarities within this virus family.

The spike protein of most nidoviruses (such as coronaviruses) is the primary target of virus-neutralising antibodies due to its role in binding to the target cell receptor [40,41]. The S protein exists as a trimer composed of monomers that contain the S1 region, forming globular heads, and a S2 region which forms a stalk that is anchored to the viral envelope [3,10]. Proteolytic cleavage of S protein in mesoniviruses is likely executed by furin [42] and, similar to some coronaviruses, this cleavage event may be necessary for receptor binding and fusion. In this context, the ability of the anti-S mAbs (C.5D3 and C.9D7 to CASV; N.6C4 and N.2E12 to NDiV) to inhibit viral infection is consistent with the neutralising capacity of anti-spike antibodies to other members of the order [10,41].

In this study mesoniviruses were detected at high frequency in populations of several mosquito species of different genera from different regions of Australia. This diversity in mosquito species suggests that, in addition to vertical transmission, other mechanisms of transmission between species may occur. Indeed, the detection of mesonivirus RNA in the saliva of wild mosquitoes via testing of FTA cards in both our study and that of others [16,19], provides evidence for horizontal transmission during mosquito feeding, although, the possibility of RNA detection from these cards resulting from the presence of infected mosquito body parts such as legs or wings, or indeed, infected excreta should be considered. However, confirmation of mesonivirus transmission via the saliva was recently provided in mosquito transmission studies [18]. The inability of mesoniviruses to replicate in vertebrate cells from a range of species suggests that a role for viral amplification in a vertebrate host is unlikely and thus, it is more likely that oral infection of mosquitoes occurs via co-feeding of an infected mosquito on the same source, whether that be virus-contaminated nectar or the simultaneous feeding on the same vertebrate [43,44,45].

## 5. Conclusions

In conclusion, we report the isolation of Alphamesoniviruses 1 and 4 from multiple mosquito species and diverse locations within Australia. Our development of mAbs against mesoniviruses provides new reagents to facilitate further research into this intriguing family of viruses. Furthermore, the antigenic studies performed herein add strength to current taxonomic classifications and current species demarcations.

## Figures and Tables

**Table 1:** Isolation of Casuarina virus (CASV) and Nam Dinh virus (NDiV) from mosquito species collected in Australia.

Species	Region	Collection Date	Mosquito Species	Pool Size	Mesonivirus Detections/Total Pools Screened
Alphamesonivirus 1 (NDiV)	Peel Region, WA	2014	*Aedes hesperonotius*	2	1/2 (incl. isolate DC59899)
			*Aedes camptorhynchus*	5–20	28/54 (incl. isolate DC60042)
			*Aedes alboannulatus*	2–13	3/4
			*Aedes notoscriptus*	1–4	2/2
			*Ae. vigilax*	2–7	2/2
			*Anopheles annulipes*	1–20	4/6
			*Cx. annulirostris*	2–5	2/2
			*Culex australicus*	6–20	4/5
			*Culex globocoxitus*	16–21	4/7
			*Culex quinquefasciatus*	8–20	3/3
			*Aedes turneri*	1	1/1
			*Cule* spp.	1	1/2
			*Aedes* spp.	21	1/1 (incl. isolate DC59801)
	Darwin, N	2018	*Culex quinquefasciatus*	1–37	2/10
			*Culex pullus*	1–47	1/14
	Ballina, NSW	2013	*Aedes notoscriptus*	25	1/3 (incl. isolate 179853)
	Brisbane, QLD	2005	*Cx. annulirostris*		1/59
			Pooled *Cx. annulirostris* and *Cq. xanthogaster*		7/100
Alphamesonivirus 4 (CASV)	Cairns, QLD	2005–2007	*Cx. annulirostris*	1–188	8/106
	Shoalwater Bay	2019	*Cx. annulirostris*		1/61
			*Ae. vigilax*		1/82
			*Ae. procax*		1/42

**Table 2:** Nucleotide identities over full genome and amino acid identities for complete ORF1ab.

	HOUV	DC59899	DC59801	DC60042	179853	NDiV	CavV	DkNV	BBaV	KSaV	KPhV	DKNV	HanaV	SWBTA-V	SWBTA-A	CASV	NSeV	KADV	OFAV	MenoV	Yichang
HOUV		***99***	***99***	***99***	***99***	***99***	***93***	***93***	***78***	***79***	***80***	***81***	***84***	***78***	***78***	***78***	***67***	***59***	***60***	***56***	***37***
DC59899	*98*		***100***	***100***	***99***	***99***	***93***	***92***	***78***	***79***	***80***	***81***	***84***	***78***	***78***	***78***	***67***	***59***	***60***	***56***	***37***
DC59801	*98*	*100*		***100***	***100***	***99***	***93***	***92***	***78***	***79***	***80***	***81***	***84***	***78***	***78***	***78***	***67***	***59***	***60***	***56***	***37***
DC60042	*98*	*99*	*100*		***100***	***99***	***93***	***92***	***78***	***79***	***80***	***81***	***84***	***78***	***78***	***78***	***67***	***59***	***60***	***56***	***37***
179853	*98*	*99*	*99*	*99*		***99***	***93***	***92***	***78***	***79***	***80***	***81***	***84***	***78***	***78***	***78***	***67***	***59***	***60***	***56***	***37***
NDiV	*98*	*99*	*99*	*99*	*99*		***93***	***92***	***78***	***79***	***80***	***81***	***84***	***78***	***78***	***78***	***67***	***59***	***60***	***56***	***37***
CavV	*91*	*91*	*91*	*91*	*91*	*91*		***91***	***77***	***78***	***80***	***80***	***83***	***78***	***78***	***78***	***67***	***59***	***60***	***55***	***37***
DkNV	*90*	*90*	*90*	*90*	*90*	*90*	*89*		***77***	***78***	***80***	***80***	***83***	***78***	***78***	***78***	***67***	***59***	***60***	***56***	***37***
BBaV	*79*	*79*	*79*	*79*	*79*	*79*	*78*	*78*		***91***	***84***	***84***	***75***	***71***	***71***	***72***	***63***	***56***	***57***	***53***	***37***
KSaV	*79*	*79*	*79*	*79*	*79*	*79*	*78*	*78*	*91*		***85***	***84***	***75***	***71***	***71***	***72***	***63***	***56***	***57***	***53***	***37***
KPhV	*81*	*81*	*81*	*81*	*81*	*81*	*80*	*81*	*82*	*82*		***96***	***77***	***74***	***74***	***74***	***65***	***58***	***58***	***54***	***37***
DKNV	*82*	*82*	*81*	*81*	*82*	*82*	*81*	*81*	*82*	*82*	*95*		***77***	***74***	***74***	***74***	***65***	***58***	***59***	***54***	***37***
HanaV	*83*	*83*	*83*	*83*	*83*	*83*	*82*	*83*	*75*	*76*	*78*	*78*		***76***	***76***	***85***	***66***	***58***	***59***	***55***	***37***
SWBTA-V	*77*	*77*	*77*	*77*	*77*	*77*	*77*	*77*	*71*	*72*	*74*	*74*	*76*		***99***	***91***	***66***	***58***	***60***	***55***	***37***
SWBTA-A	*77*	*77*	*77*	*77*	*77*	*77*	*77*	*77*	*71*	*72*	*74*	*74*	*76*	*98*		***90***	***66***	***58***	***60***	***55***	***37***
CASV	*77*	*77*	*77*	*77*	*77*	*77*	*77*	*77*	*71*	*71*	*74*	*74*	*76*	*96*	*95*		***65***	***58***	***59***	***55***	***37***
NSeV	*69*	*69*	*69*	*69*	*69*	*69*	*69*	*69*	*65*	*66*	*67*	*68*	*68*	*67*	*67*	*68*		***59***	***59***	***55***	***37***
KADV	*61*	*61*	*61*	*61*	*61*	*61*	*61*	*62*	*59*	*59*	*61*	*61*	*61*	*61*	*61*	*61*	*62*		***60***	***57***	***37***
OFAV	*58*	*58*	*58*	*58*	*58*	*58*	*58*	*59*	*56*	*56*	*58*	*58*	*59*	*57*	*58*	*58*	*58*	*59*		***56***	***37***
MenoV	*63*	*63*	*63*	*63*	*63*	*63*	*63*	*63*	*60*	*60*	*62*	*62*	*62*	*62*	*62*	*62*	*62*	*61*	*57*		***36***
Yichang	*46*	*46*	*46*	*46*	*46*	*47*	*46*	*47*	*46*	*47*	*47*	*47*	*46*	*46*	*46*	*46*	*46*	*45*	*45*	*45*	

Bold text: amino acid identity; non-bolded text: nucleotide identity.

**Table 3:** Reactivity of mAbs towards CASV and NDiV in ELISA.

Name	Made to	Isotype	Cross-Reactivity *	Protein Target ^#^	Microneutralisation ^§^
C.1G9	CASV	IgG1	−	N	<1
C.5D3	CASV	IgG2a	−	S/S1	>128
C.5F8	CASV	IgM	+	M	<1
C.6B9	CASV	IgM	+	M	<1
C.8G3	CASV	IgG1	−	N	<1
C.9D7	CASV	IgG2a	−	S/S2	>128
C.10C1	CASV	IgG2b	−	M	<1
C.10H11	CASV	IgM/IgG2a	+	M	<1
N.1A10	NDiV	IgM	−	−	<8
N.1E6	NDiV	IgM	−	−	16
N.2A11	NDiV	IgG2a	+	M	<8
N.2B2	NDiV	IgM	+	M	<8
N.2D10	NDiV	IgG2a	+	−	<8
N.2E12	NDiV	IgG2b	−	S1	>1024
N.2G8	NDiV	IgM	−	−	8
N.3A10	NDiV	IgG2a	−	−	16
N.3B2	NDiV	NT	−	ND	8
N.3B11	NDiV	IgG3	+	M	<8
N.3C10	NDiV	IgM	−	N	8
N.3D9	NDiV	IgG1	+	−	128
N.3G4	NDiV	IgG3	+	M	<8
N.4C11	NDiV	IgM	+	ND	<8
N.4D3	NDiV	IgM	+	M	<8
N.4F9	NDiV	IgM	−	−	<2
N.4H1	NDiV	IgG3	−	N	<2
N.4H7	NDiV	IgG1	−	N	<2
N.5E10	NDiV	IgM	+	ND	<2
N.6A3	NDiV	IgG1	−	−	<8
N.6B3	NDiV	IgG1	−	−	<8
N.6C3	NDiV	IgG1	−	−	<2
N.6C4	NDiV	IgG2a	−	S	>256
N.6F2	NDiV	IgG2a	+	−	16
N.6G5	NDiV	IgM	−	−	<2
N.7C7	NDiV	IgG1	−	−	<8

* Positive reactivity was based on assessment of the mAb on the reciprocal virus and produced an absorbance value (at OD405 nm) of >0.25 upon subtraction of the value for uninfected cells. ^#^ Determined by Western blot or immunoprecipitation. − indicates that the sample was not reactive in Western blot. ^§^ Neutralisation assay results against homologous virus. Values are the reciprocal of highest mAb dilution at which 100 infectious units of virus was neutralised. S: spike protein, M: membrane protein, N: nucleocapsid protein, NT: not tested, ND: not determined. These mAbs detected proteins in Western blot that could not be differentiated between M and N.

**Table 4:** Location and collection date of FTA cards tested for mesonivirus RNA by RT-PCR.

Location	Collection Year	Samples Positive/Tested for Mesonivirus RNA
Emerald	2012/13	1/8
Cape York	2012/14	0/8
Mt Isa	2012	0/1
Darwin	2012	2/8
Rockhampton	2013	0/8
Townsville	2013	0/10
St George	2013	0/1
Charleville	2013	0/1
Mareeba	2013	0/1
Badu Island	2013	0/2
Seisia	2013	0/1
Longreach	2013	4/8
		7/57

## Supplementary Materials

The following are available online at https://www.mdpi.com/1999-4915/12/10/1159/s1, Figure S1. Mosquito trapping locations within Australia where mesoniviruses were detected; Figure S2. Western blot analysis of CASV-reactive mAbs; Figure S3. Immunoprecipitation analysis of CASV-reactive mAbs; Figure S4. Sequencing read coverage following RNA elution from FTA card. Table S1. Reactivity of CASV-reactive mAbs towards CASV and NDiV in ELISA.
