# Supplementary material for: Genetic, Morphological and Antigenic Relationships between Mesonivirus Isolates from Australian Mosquitoes and Evidence for Their Horizontal Transmission
Source: Viruses. 2020 Oct 13;12(10):1159. doi: 10.3390/v12101159 (PMC7602028; doi:10.3390/v12101159)
Supplement: Supplementary file 1 [file viruses-12-01159-s001.pdf]

Supplementary Data

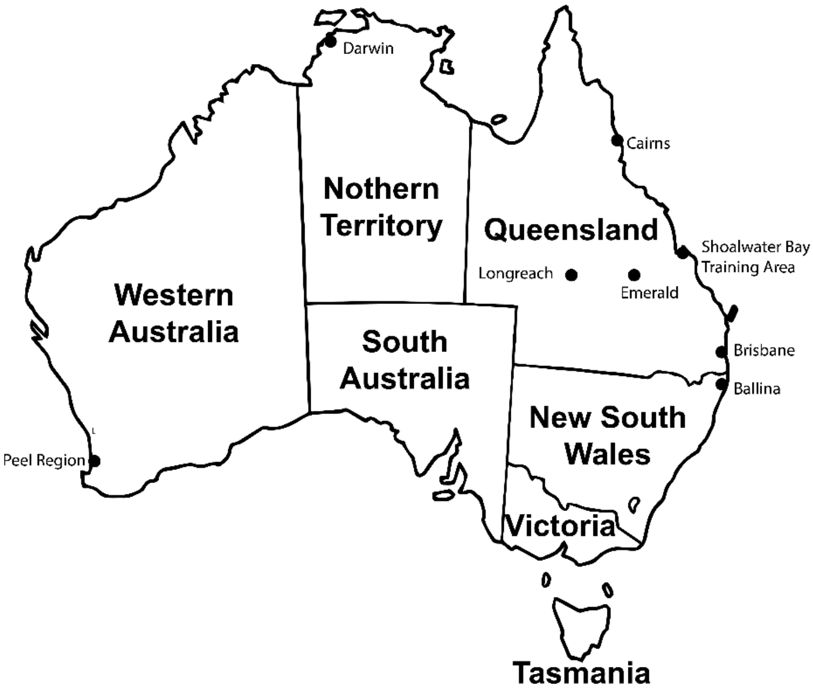

Figure S1. Mosquito trapping locations within Australia where mesoniviruses were detected.

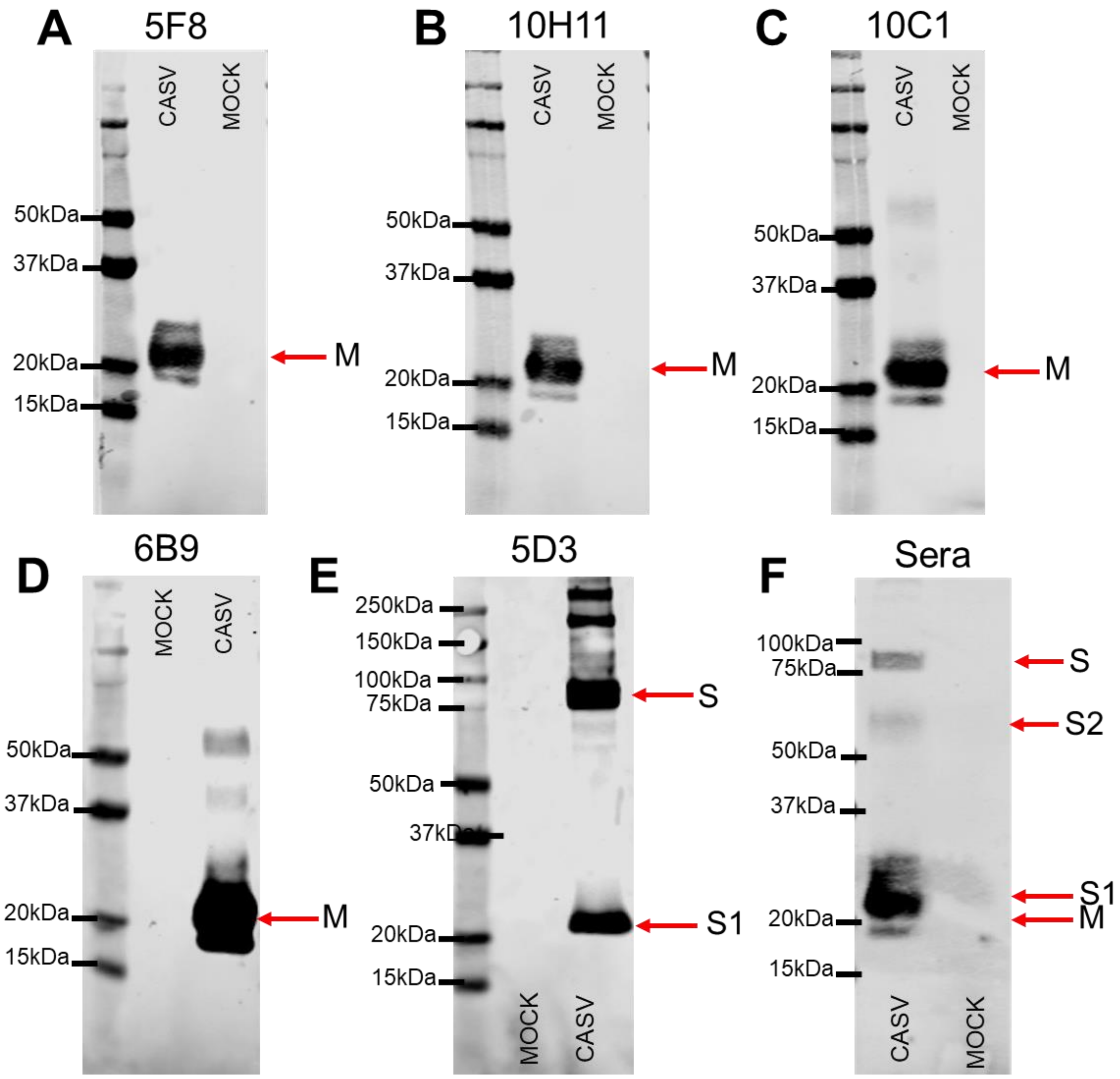

Figure S2. Western blot analysis of CASV-reactive mAbs. A lysate of CASV-infected C6/36 cells was resolved by SDS-PAGE on 4-12% Bis-Tris gels and transferred to nitrocellulose membrane. The proteins were probed with anti-CASV mAbs (panels **A**–**E**), or with anti-CASV polyclonal mouse serum (**F**). Predicted proteins are indicated with arrows.

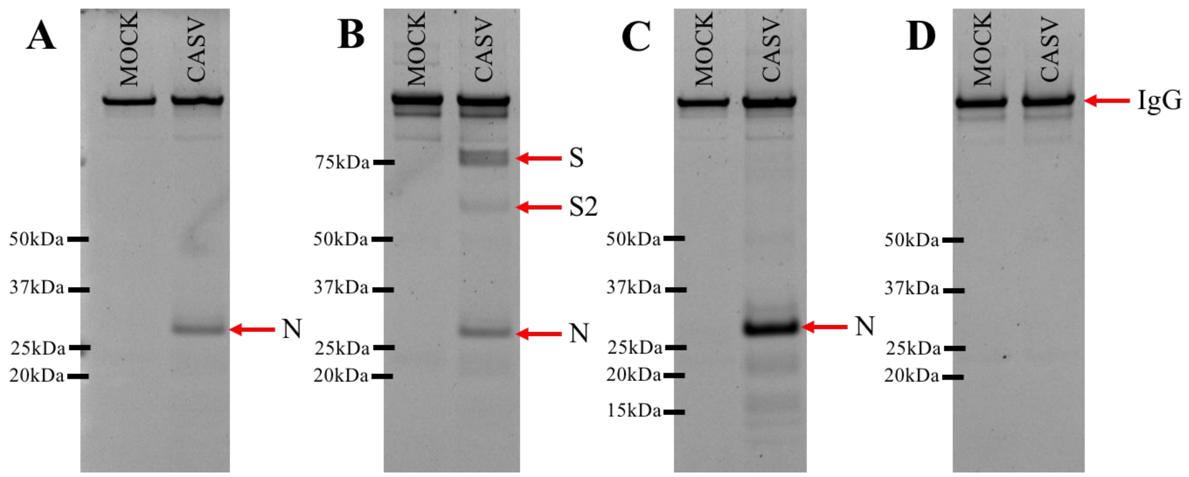

Figure S3. Immunoprecipitation analysis of CASV-reactive mAbs. Anti-CASV mAbs bound to Protein G beads were used to precipitate CASV proteins from a lysate of CASV-infected C6/36 cells. Immunoprecipitated proteins were resolved by SDS-PAGE gel electrophoresis and visualised by total protein staining (Sypro Ruby). (**A**) mAb C.8G3, (**B**) mAb C.9D7, (**C**) mAb C.1G9 and (**D**) isotype control mAb 4G2 (anti-flaivivirus E).

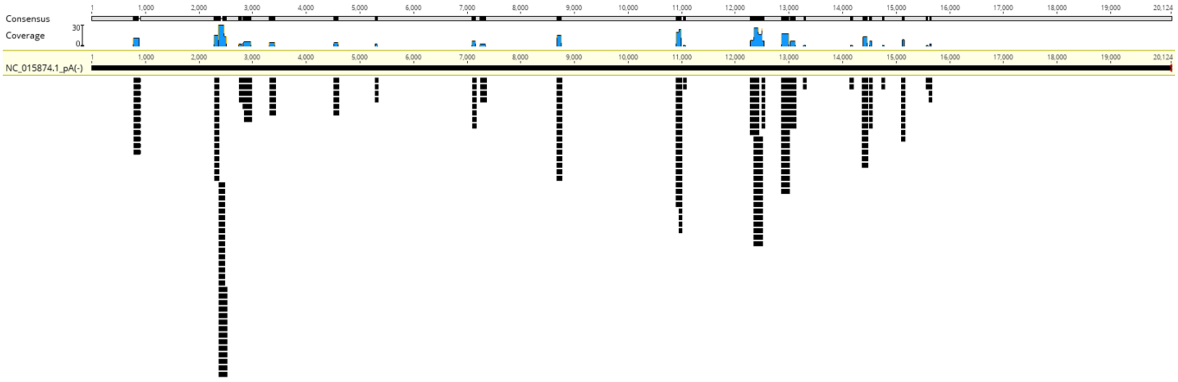

Figure S4. Sequencing read coverage following RNA elution from FTA card. Next generation sequencing was performed on RNA eluted from a honey-baited FTA card. Coverage of the genome was determined by reference assembly against the NDiV genome as a reference (GenBank accession number NC_015874.1).
